# Flexible compensation of uniparental care: female poison frogs take over when males disappear

**DOI:** 10.1093/beheco/arv069

**Published:** 2015-05-29

**Authors:** Eva Ringler, Andrius Pašukonis, W. Tecumseh Fitch, Ludwig Huber, Walter Hödl, Max Ringler

**Affiliations:** ^a^Department of Integrative Zoology,; ^b^Department of Cognitive Biology, University of Vienna, Althanstrasse 14, A-1090 Vienna, Austria, and; ^c^Messerli Research Institute, University of Veterinary Medicine Vienna, Medical University of Vienna and University of Vienna, 1210 Vienna, Austria

**Keywords:** behavioral flexibility, compensation, partner removal, poison frog, tadpole transport, uniparental care.

## Abstract

Parental care systems are shaped by costs and benefits to each sex of investing into current versus future progeny. Flexible compensatory parental care is mainly known in biparental species, particularly where parental desertion or reduction of care by 1 parent is common. The other parent can then compensate this loss by either switching parental roles and/or by increasing its own parental effort. In uniparental species, desertion of the caregiver usually leads to total brood loss. In the poison frog, *Allobates femoralis*, obligatory tadpole transport (TT) is generally performed by males, whereas females abandon their clutches after oviposition. Nevertheless, in a natural population we previously observed 7.8% of TT performed by females, which we could link to the absence of the respective fathers. In the following experiment, under laboratory conditions, all tested *A. femoralis* females flexibly took over parental duties, but only when their mates were removed. Our findings provide clear evidence for compensatory flexibility in a species with unisexual parental care. Contrary to the view of amphibian parental care as being stereotypical and fixed, these results demonstrate behavioral flexibility as an adaptive response to environmental and social uncertainty. Behavioral flexibility might actually represent a crucial step in the evolutionary transition from uniparental to biparental care in poison frogs. We suspect that across animal species flexible parental roles are much more common than previously thought and suggest the idea of a 3-dimensional continuum regarding flexibility, parental involvement, and timing, when thinking about the evolution of parental care.

## Lay Summary

Caring mothers step in for deadbeat dads. Flexible compensation has evolved as a countermeasure against reduced or lost parental care and is commonly found in biparental species. In the poison frog *Allobates femoralis* with obligatory male-only care, we show that females flexibly perform tadpole transport when males disappear. This demonstrates that compensatory flexibility also evolved in species with unisexual care, suggesting that parental care systems are more flexible than previously thought.

## INTRODUCTION

In species, where one or both parents provide care for their offspring, the actual parental investment of both sexes is shaped by the trade-off between current and future mating opportunities (Trivers 1972). Parental behavior of males and females is expected to coevolve, as the behavior of each sex impacts the fitness of the other (Lessels 2012). When both parents are involved in brood care, males and females cooperate, often by exhibiting different parental roles. In several species, sex-specific parental roles are physiologically determined in one sex (e.g., female lactation in mammals, male brood pouches in sea horses) or may have become stabilized due to behavioral and environmental constraints over evolutionary time (Clutton-Brock 1991; Klug *et al.* 2012). Otherwise, parental roles can also be negotiated between the partners after mating, up to the point where only one of the partners is left to care for the offspring (i.e., amphisexual parental behavior sensu Simon 1983).

Theoretical models predict that a change in the intensity or frequency of parental care by 1 parent should lead to a behavioral change in the other parent at both evolutionary (Houston and Davies 1985) and behavioral timescales (McNamara *et al.* 2003). Thus, in species where sex-specific parental roles have evolved, the loss of one parent, either because of active brood desertion or due to death, might lead to a spontaneous change of the sex-specific caring behavior in the remaining parent. Such behavioral flexibility in parental care is currently known almost exclusively in biparental species, especially where parental desertion or care reduction by 1 parent is relatively common. In several biparental fish, insects and birds, it has been shown that experimentally widowed parents are capable of raising offspring alone, either by increasing their usual parental activities or by altering their parental behavior (Itzkowitz *et al.* 2001; Harrison *et al.* 2009; Suzuki and Nagano 2009). These studies corroborate theoretical models suggesting that the roles displayed in biparental species may be flexible and depend on the presence and the behavior of the other parent. Many studies of parental care have investigated variation in the behavior of entire populations across environments, whereas little is known about behavioral flexibility within individuals (Royle *et al.* 2014).

A modified form of parental flexibility is found in amphisexual species (Simon 1983), where parental care is provided by either the male or the female, but not by both. In these species, parental duties are not fixed, but instead are established directly after oviposition (see also Michiels 1998 for mating conflicts and sperm competition in hermaphrodites). Either sex may care for the offspring, depending on which parent manages to desert first in the attempt to remate and sire/lay another clutch. In the Penduline Tit (*Remiz pendulinus*), for example, the behavior of females is affected by the male decision to desert or stay, immediately after oviposition, demonstrating that the female’s reproductive tactics are flexible (Czyż 2011). Similar behavior is also known in several frog species (Bourne 1998; Lehtinen 2003). In contrast to amphisexual behavior where parental roles are engaged directly after oviposition, we consider “true” spontaneous flexibility as behavior that is employed only after the initial parental roles have been established, and at any given time during care.

In uniparental species, desertion or death of the sole caregiver often leads to high or complete offspring mortality (Eggert *et al.* 1998, Lehtinen *et al.* 2014). Thus, one should expect major fitness benefits of compensatory parental behavior for both sexes, increasing with the risk of parental desertion and with decreasing costs of care. However, spontaneous initiation of parental care by the noncaring sex as a response to the loss of the former caregiver is extremely rare (or at least little known) in species with unisexual parental care, and was so far only observed in some group-living arthropods that occur at rather high densities (e.g., assassin bugs: Beal and Tallamy 2006; harvestmen: Buzatto and Machado 2009). However, in these species, males and females form polygynous harems where the noncaring sex remains in very close proximity (e.g., inside the territory) to the caregiver during the entire period of parental care.

The dendrobatid frog *Allobates femoralis* is a small ground-living rainforest frog whose behavior, population genetics, ecology, and biogeography are well studied (Roithmair 1992; Narins *et al.* 2005; Simões *et al.* 2008; Amézquita *et al.* 2009; Ursprung *et al.* 2011; Ringler *et al.* 2013). During the reproductive season, males call from elevated structures like logs or roots on the forest floor to announce territory possession to male competitors and to attract females, who stay at perches up to 20 m outside the males’ territories and show nonaggressive site fidelity (Ringler *et al.* 2009, 2012). Pair formation, courtship, and mating without amplexus (i.e., the male cannot force the female to mate by clasping her), take place inside the males’ territories, where externally fertilized clutches of approximately 20 eggs are laid in the leaf litter. After oviposition females immediately abandon the clutch and return to their perches outside the males’ territories (Roithmair 1992; Ringler *et al.* 2009; Montanarin *et al.* 2011; Ringler *et al.* 2012). Under optimal conditions, females can ovulate and produce a clutch every 8 days (Weygoldt 1980, in captivity). Thus, during the 3 weeks of larval development of a clutch females may have produced up to 3 further clutches. Usually, these clutches are sired by different males (Ringler *et al.* 2012), as both sexes are highly polygamous during the prolonged reproductive periods (Ursprung *et al.* 2011). Males were observed to attend to up to 5 clutches simultaneously (Ursprung *et al.* 2011).

The only known expression of parental care in this species is the obligate transport of tadpoles to water after 15–20 days of larval development, which is generally performed by the father. Tadpoles that are not transported cannot finish their development inside the clutch and die (Weygoldt 1980; Ringler *et al.* 2013; E Ringler, M Ringler unpublished data). Sporadic observations of females with larvae on their back (Crump 1974; Silverstone 1976; Caldwell and Araújo 2005) have led previous authors to describe *A. femoralis* as biparental (cf. Grant *et al.* 2006; Wells 2007). However, this conjecture was based purely on anecdotal reports and was never investigated systematically. A recent study on patterns of tadpole transport (TT), conducted over 5 years in a natural population of *A. femoralis* in French Guiana, reported 7.8% of TT (10 out of 129) by females (Ringler *et al.* 2013, Figure 1). Here, we hypothesize that *A. femoralis* actually is a uniparental species with general male care and that occasional female TT has evolved as a flexible compensation of lost male care to insure against total offspring loss.

**Figure 1:**
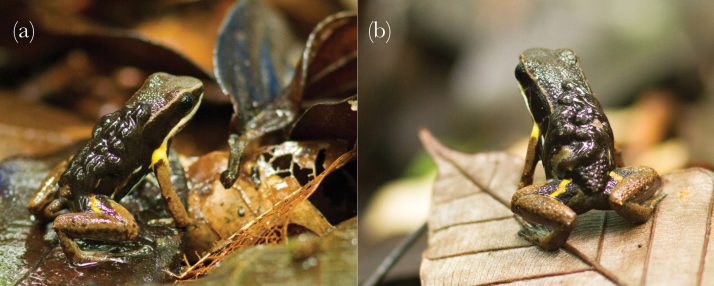
TT of *Allobates femoralis* in the field. (a) male, (b) female.

## METHODS

We performed a spatio-temporal reanalysis of 5 years of field data and parentage assignments from the study of Ringler *et al.* (2013) on TT in a natural population of *A. femoralis*. Monitoring took place in a natural *A. femoralis* population located near the field camp “Saut Pararè” (4°02′N, 52°41′W) in the nature reserve “Les Nourages,” French Guiana during their reproductive period from 2008 to 2012. We aimed at a total sampling with estimated sampling coverages of on average 76.83% and 56.38% for males and females, respectively, in the study plot (cf. Ringler *et al.* 2015). We recorded the precise spatial locations of all frogs in the field on a digital map (cf. Ringler *et al.* 2014) and added behavioral observations, such as notes on TT activity and courtship. For this study, we reanalyzed the entire capture histories of those fathers whose tadpoles were sampled during transport (by females and by males). We checked if the males were observed (i.e., sampled by us) 1) at least once within 3 weeks prior to TT, 2) after TT, 3) before and afterward, or 4) if their only capture occurred during TT. We then tested whether inferred fathers of the tadpoles which had been transported by females disappeared significantly more often from the sample than fathers that had carried the tadpoles themselves. Based on the individual capture histories, we assessed which fathers disappeared after the transport, 3 weeks prior to TT, and which fathers had just been recorded during TT. We used a 2-proportions *Z*-test to determine whether the difference between the ratios of fathers that disappeared was significant between groups.

In a second step, we tested experimentally whether female TT behavior is elicited by male absence. Therefore, we performed a behavioral experiment under controlled laboratory conditions from October 2012 to October 2013 in the animal care facilities at the University of Vienna with wild-caught *A. femoralis* from French Guiana. Permissions for sampling and exportation of animals were obtained from the responsible French authorities (DIREN: Arrete n° 82 du 10 August 2012 & Arrete n° 4 du 14 January 2013). We used standard glass terraria of equal size (60cm × 40cm × 40cm) with identical equipment and furnishings. The floor was filled with pebbles of expanded clay, back and side walls were covered with Xaxim (tree fern stems) mats in the lower and cork in the upper half to prevent visual contact between terraria. Half a coconut shell, a small plant, and a branch provided standardized shelter and elevated calling positions. We provided oak leaves as substrate for oviposition, and a small glass bowl of 10-cm diameter filled with water for tadpole deposition. An automatic raining, heating, and lighting system ensured standardized climatic conditions with equal parameters to the natural conditions in French Guiana, in all terraria. Prior to our experiments, all males and females were arbitrarily kept in pairs during several months, and had already produced multiple clutches with multiple partners. During this time, we only observed males performing TT and not a single case of TT by females.

In preliminary trials, we permitted pairs (*N* = 9) to mate and produce 1 clutch. After oviposition, males were experimentally removed (approximately 5 days after oviposition), and we observed whether the females performed TT to the water body available inside the terrarium. We then paired 15 *A. femoralis* males and females, respectively, until each pair had produced at least 2 clutches. Both parents were initially left in the terrarium and we recorded which parent transported the larvae of the early clutch. Afterward, the first clutch got transported, the males were removed from the experimental terraria and we recorded the subsequent behavior of the females. Because in the preliminary trials, all females already carried the tadpoles of the “first” (and only) clutch after male removal, and in the main experiment they showed the same reliable behavior towards their “second” clutch (see Results), we ruled out that this behavior was actually an effect of clutch order. We therefore did not perform an additional control with males not removed after the first TT.

Males and females were paired to optimize visual differentiation by means of the individuals’ lateral coloration patterns in order to avoid captures for identification. In a few cases where the identification was ambiguous, we captured the individual only when it was already carrying tadpoles on its back. As the whole process from tadpole uptake until release usually lasted several hours and tadpole deposition always occurred in the morning, hourly monitoring of transportation activity was sufficient to observe all transporting events.

## RESULTS

In the natural population of *A. femoralis*, we observed 119 TT events by males and 10 cases of transport by females over 5 years (Ringler *et al.* 2013). After the transport, fathers of the tadpoles which had been transported by females disappeared significantly more often from the sample than fathers that had carried the tadpoles themselves (2-proportions *Z*-test, *Z* = 2.3605, *P* = 0.018; Table 1). Furthermore, significantly more fathers of tadpoles carried by females were not recorded during 3 weeks prior to TT (*Z* = 2.915, *P* = 0.004), not recorded before and after (*Z* = 2.314, *P* = 0.021), or not at all (*Z* = −3.138, *P* = 0.002).

**Table 1:** Presence of fathers from tadpoles transported by males and females in the field

Father observed at least once	Transporting parent	
	Male (*N* = 119)	Female (*N* = 10)
Three weeks before TT	79 (66.4%)	2 (20%)
After TT	80 (67.2%)	3 (30%)
Before and after TT	57 (47.9%)	1 (10%)
Never	15 (12.6%)	5 (50%)

TT: tadpole transport; “Never” includes fathers that were only observed during TT (male TT events) as well as fathers that were never sampled in the course of our study (simulated paternal genotypes in female TT events); categories are not exclusive.

In captivity, male and female *A. femoralis* showed behavior similar to prior observations in the field: males called from elevated positions; as soon as a female was close, her presence elicited courtship calls and initiated courting behavior; and clutches were deposited in the leaf litter. Beside courtship and mating, there were no further apparent interactions between males and females, and especially no aggression between the partners. All TT was performed during morning hours.

**Figure 2:**
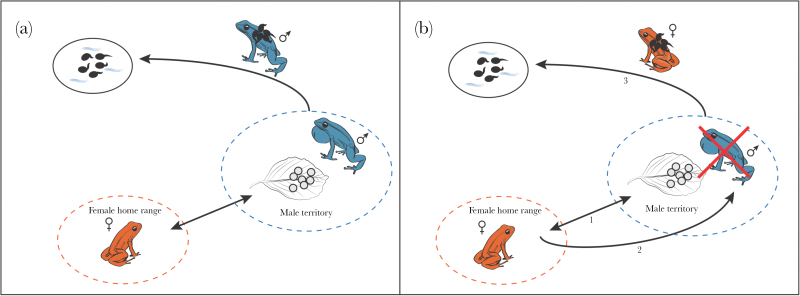
TT in *A. femoralis*. (a) Regular pattern of male TT: females approach a nearby calling male, eggs are laid inside the male territory, females home back to their resting sites, after about 3 weeks of larval development males carry the tadpoles to water pools. In our experiments, males performed TT in all cases (*N* = 15) where both parents were present inside the terrarium. (b) Flexible compensation of missing paternal care by females: when the male was experimentally removed after oviposition from the terrarium, all tested females (*N* = 15) spontaneously performed TT. Numbers indicate the sequence of female movement.

In the preliminary trials, males were removed about 5 days after oviposition (mean ± SD = 4.56±1.71 days), and all females performed TT as soon as the tadpoles hatched (mean ± SD = 18.56±2.87 days after oviposition). In the main experiment, the time between first and second clutch was about 2 weeks (mean ± SD = 16.93±6.65 days). In all cases (*N* = 15), the tadpoles of the first clutch were transported by the fathers (Figure 2a, Table 2), whereas the female was present in the terrarium. During the development of the clutches, we did not observe females caring for or checking the clutches. Males never reacted aggressively toward the females in the terraria. After males had been removed from the experimental terraria, in all cases the mothers transported the larvae of the second clutch (*N* = 15, Figure 2b, Table 2). Overall, female TT took place on average 21 days after oviposition, and this delay was not significantly different between males and females (males: mean ± SD = 20.53±2.07 days, females: mean ± SD = 20.87±3.54 days; *t*-test: *t* = −0.315, df = 28, *P* = 0.755).

**Table 2:** TT behavior during the male-removal experiment

TT	Males	Females
First clutch	15/15	0/15
Second clutch	n/a	15/15

First clutch: male and female present inside the terrarium; Second clutch: female present, male removed. n/a, not applicable.

## DISCUSSION

Our findings provide clear evidence for compensatory flexibility in a uniparental species with generally fixed sex-specific parental roles. The spatio-temporal analysis of the field data (Ringler *et al.* 2013) revealed that in 90% of the cases of female TT the respective father was not available for transporting at the time when the tadpoles had finished their intraclutch development approximately 3 weeks after oviposition. Only one single father was recorded before and also after the transport, but he had performed a territory shift the week before the observed female TT event. Based on the documented behavior of *A. femoralis* females to abandon the clutch immediately after oviposition and to return to their resting sites which are up to 20 m distant from male territories, we preclude negotiated amphisexual care sensu Simon (1983). In our study, the flexible compensatory behavior is not conducted immediately after oviposition during the establishment of parental roles, but instead is initiated much later and only in cases when the male disappears during the period of clutch development, which takes about 3 weeks. We conclude that the rare observations of female TT in *A. femoralis* in the field are the result of flexible compensatory behavior in response to male absence and not a manifestation of general biparental care.

### Mechanisms of female care compensation

In our experiment, all tested *A. femoralis* females refrained from performing TT whenever the males were present, but in all cases they flexibly took over parental duties when the males were removed. Females were not prevented from approaching the clutches by the fathers, and males with clutches either acted indifferently toward females or kept on courting them to acquire further clutches. This contrasts seemingly similar reports of compensatory female care in species with general male care, where females showed a strong tendency to stay with or return to the clutch, but were forced away by their mates (Myers and Daly 1983; Bourne 1998). Our field observations on male presence/absence and female TT strongly suggest that the findings from captive animals will generalize to behavior in the wild.

Considering only the previous field observations, TT by females could have multiple alternative explanations: 1) females routinely return to their clutch sites 3 weeks after oviposition—or slightly later—and take up the tadpoles in case the male has not yet transported the clutch; 2) some females have similar hormonal states as males and therefore act like males regarding parental care; 3) some females occasionally stay inside the male’s territory and do not return to their resting site and then also perform TT; (4) females transport any/all hatched tadpoles they discover by chance. Previous studies as well as our present experiment, however, demonstrate that temporal patterns of TT were not significantly different between males and females and that female presence did not deter males from transporting the tadpoles. We elicited TT in all tested *A. femoralis* females, but only as a response to male removal. We therefore argue that this behavior is unlikely to be the result of exceptional hormonal states or unusual spatial behavior of specific females. In *A. femoralis*, females do not stay close or routinely return to a clutch a few days after oviposition (Roithmair 1992; Montanarin *et al.* 2011; Ringler *et al.* 2013), so chances are low for them to accidentally encounter clutches ready to be transported. In the natural *A. femoralis* population studies by Ringler *et al.* (2013), maternity was assigned in all cases to the carrying female. In our experiment, females did not transport clutches as long as the male was present, although the fathers did not hinder them from approaching the clutches. This excludes the alternative explanation that females might transport any clutch they encounter (including unrelated ones).

One of the fundamental prerequisites for behavioral flexibility is to properly detect, identify, and adequately respond to changes in the environment. Our finding that females flexibly take over TT raises the question of how they detect the absence of their former mating partners under natural conditions, over considerable distances, typically up to 20 m (Ringler *et al.* 2012), in dense rainforest. As vocal communication plays a key role in social interactions, such as courtship, mating, and territorial defense in amphibians, we hypothesize that acoustic cues enable females to monitor the presence of males. Male *A. femoralis* typically broadcast their prominent advertisement call over several hours throughout the day (Hödl 1983), in order to repel rivals as well as to attract mates. Consequently, females may take advantage of this high male calling activity to acoustically monitor male presence at the calling site. Ceased advertisement calls of a male over a longer period of time could be the result of the male’s death, a territory take-over by another male or brood desertion for unknown reasons. As a compensatory response, females might initiate TT behavior in order to avoid total clutch loss.

To perform TT, females need to remember the exact location of clutches laid about 3 weeks before, which strongly suggests the existence of long-term spatial memory. This is consistent with the results of telemetry studies in the field which revealed sophisticated orientation behavior in *A. femoralis* males (Pašukonis *et al.* (2014a)), but only when they were translocated within familiar areas (Pašukonis *et al.* (2014b)). Given that females can ovulate and produce a clutch every 8 days (Weygoldt 1980, in captivity) and larval developmental time inside clutches is approximately 3 weeks, females may have to simultaneously remember and monitor up to 4 clutch locations and males, respectively. Further experimental studies are needed to identify which cues are actually used by females to initiate their flexible parental behavior.

### Parental strategies of males and females

Theories on parental decision making suggest that such compensatory behavior by *A. femoralis* females could be exploited by males through clutch desertion (cf. Székely *et al.* 1996, Barta *et al.* 2002). Sexual conflict is a central driver in the evolution of parental care (Trivers 1972, Parker 1979, Gross and Sargent 1985, Gross 2005) and has led to various antagonistic or cooperative behaviors between the sexes (Chapman *et al.* 2003). In general, each sex should prefer that the other provide (more) care, in order to be able to reduce own parental efforts (Westneat and Sargent 1996, Royle *et al.* 2002). Males could coerce females into performing TT in several different ways. On one hand, they could leave the clutch and their territory to settle somewhere else. However, this would mean abandoning a “good” territory—one in which they already obtained at least one successful mating. Given the high male territoriality and iteroparity in *A. femoralis* throughout the prolonged reproductive period (Ursprung *et al.* 2011), the costs of establishing a new territory after every clutch are likely to greatly exceed the costs of parental care. By the same logic, an attempt to “trick” females into care by ceasing calling activity would entail abandoning the territory, given the prominent role of vocalization in territory defense. If the male simply failed to transport the tadpoles, the female would have to actively check the presence of the male herself repeatedly to detect the coercion—a behavior never observed so far. All these potential strategies thus are likely to be detrimental to male fitness in terms of lost mating opportunities and/or reduced offspring survival; costs which will not be outweighed by the reduced costs of less care.

Considering the high degree of male territoriality and the iteroparity in this species, we suggest that male parental care may be an evolutionarily stable strategy in *A. femoralis*. Similar factors have probably driven the evolution of male-only care in fish, which is a common form of parental care in this large taxon (cf. Gross 2005). For female *A. femoralis*, in turn, the optimal strategy is more likely to be a mixed one (cf. Maynard Smith and Parker 1976), where they typically leave parental care to males and only initiate parental behavior in cases of mate loss in order to avoid total clutch loss. Consequently, we regard the observed flexibility not as a behavioral response to “active” male desertion, but rather as an evolutionary “backup” to partner loss.

### Costs and benefits of care compensation

In *A. femoralis*, the direct costs of TT, such as energy expenditure and predation risk, are presumably similar for males and females. However, the overall costs of TT might be lower for females than for males, given that females do not defend territories and thus do not risk losing a territory during their absence. Female oviposition is not restricted to a narrow time frame because the breeding season lasts for several months and females produce multiple clutches during this period. Consequently, we suspect that occasional TT events should not severely reduce mating opportunities for *A. femoralis* females. Therefore, we conclude that females can gain substantial fitness benefits by performing parental care to compensate for mate loss and suggest that the flexibility documented here represents an evolved and adaptive response to environmental and social uncertainty.

The findings of the present study are likely to generalize to other animal taxa. By flexibly compensating for reduced or lost parental care, individuals can assure the further survival of an entire clutch in which they already invested substantial time and energy. This is particularly relevant to species where parental care is obligate for offspring survival and to short-lived species where each successful clutch substantially contributes to an individual’s reproductive success. Together with an increasing risk of lost parental care and decreasing costs of care this will favor the evolution of compensatory parental flexibility, even in species where typically only one sex performs care. Based on our findings, we strongly encourage theoretical biologists to model the evolution of compensatory behavior in parental care.

### Behavioral flexibility in amphibian parental care

Previous studies on behavioral flexibility mainly focused on mammals and birds (Kappeler and Kraus 2010), despite some evidence for behavioral flexibility in other animal taxa, including fish (Itzkowitz *et al.* 2001), reptiles (Wilkinson and Huber 2012), insects (Suzuki and Nagano 2009), and mollusks (Finn *et al.* 2009). Herpetologists have long appreciated the flexibility and context-dependent nature of amphibian behavior (e.g., see Chapters 6–11, and 12 in Wells 2007 and citations therein). However, many nonspecialists still consider frogs to be rather instinct-bound and displaying stereotypic behavior. This view is gradually changing as a result of more recent behavioral and neurophysiological studies (Dicke and Roth 2009; Burghardt 2013), and our results will contribute to this trend. Poison frogs show a remarkable complexity and diversity in spatial behavior and parental care (Wells 2007) and are a promising taxon for research on behavioral flexibility. However, little is known about such processes and abilities in this family, or indeed for amphibians in general.

### Parental care in poison frogs

The emergence of behavioral flexibility as observed in our study could constitute a step in the evolutionary transition from uniparental to biparental care in poison frogs by integrating females into a previously uniparental paternal care system. Male TT without any provisioning or attendance is proposed to be the ancestral form of parental care in dendrobatid frogs (Weygoldt 1987; Summers and Tumulty 2014). Due to the fact that clutches are laid inside male territories—probably to prevent secondary fertilizations by male opponents—and externally fertilized without amplexus, females can immediately desert and thereby ‘force’ males into parental care (Barta *et al.* 2002; Lessels 2012). Nonetheless, exclusive female or biparental care has evolved in several species (Tumulty *et al.* 2014). Most species carry their larvae from terrestrial egg-deposition sites to waterbodies such as small streams, swamps, temporary ponds, or to phytotelmata in leaf axils, bromeliads or tree holes. In some species, larvae even complete their entire development while being carried on the parent’s back (Wells 2007). Recent research on several dendrobatid species has demonstrated the presence of behavioral plasticity in deposition strategies in regard to multiple factors, such as predator presence (McKeon and Summers 2013; Schulte and Lötters 2014), pool quality (Poelman *et al.* 2013) and seasonal variation of desiccation risk (Schulte and Lötters 2013). Given several other anecdotal observations of alleged biparental care in closely related dendrobatid species (listed in Wells 2007, Table 11.3, p. 524–526; Myers and Daly 1983), this specific compensatory behavioral flexibility is probably more widespread and might have deeper evolutionary roots in dendrobatid frogs (see also Killius and Dugas 2014, Tumulty *et al.* 2014). Contrary to the current view of patterns of parental care in poison frogs as a stereotypic and stable result of long-term evolutionary processes (Weygoldt 1987; Grant *et al.* 2006; Lötters *et al.* 2007; Summers and Tumulty 2014), our experiments show that individuals of the noncaring sex are able to immediately compensate for a lack of parental care by their partner. This is of major importance for studies that try to explain evolutionary processes and phylogenies—not only in poison frogs—by comparing “defined” patterns of parental care among species.

### Classifying parental care systems

Previous classifications of parental care systems are mainly based on whether and to what extent the male, the female, or both provide care. We feel that the established distinction between uniparental and biparental care should be treated with caution, as it might be oversimplified for many animal species. We suspect that flexible parental roles might be much more common than previously thought, and in several species that are currently classified as biparental, actually uniparental care with flexible compensation might be the case. Flexibility in parental behavior—as opposed to invariable and fixed parental systems—is a more realistic expectation under often highly variable and unpredictable environmental and social circumstances. In this context, amphisexual care sensu Simon (1983) should be regarded as parental flexibility at the earliest possible stage, thereby adding a temporal component to the classification of parental care systems.

Furthermore, amphisexual care and likewise uniparental care with compensatory flexibility are special cases of both uniparental care and biparental care. With the focus on the parent pair, both systems are rather uniparental, as at any given time only one of the partners is taking care of the mutual offspring. However, on the species level, both systems can be seen as special cases of biparental care, as overall a certain percentage of care is performed by either of the sexes. Accordingly, we want to endorse the idea of a 3-dimensional continuum—from fixed roles to flexibility, from uniparental to biparental care, and from early to late assumption of parental roles—when thinking about the evolution of parental care.

## FUNDING

This study was financed by the Austrian Science Fund (FWF) through the project P24788-B22 (PI Eva Ringler) and the doctoral program “Cognition and Communication” W1234-G17. The Nouragues Ecological Research Station is supported by the CNRS.
